# Pharmaco-chemical profiling of *Desmodium gangeticum* (L.) DC. with special reference to soil chemistry

**DOI:** 10.1186/s43094-021-00356-7

**Published:** 2021-10-17

**Authors:** Prasobh K. Mohan, T. P. Adarsh Krishna, T. Senthil Kumar, B. D. Ranjitha Kumari

**Affiliations:** 1 Department of Botany, Bharathidasan University, Tiruchirappalli, Tamil Nadu 620 024 India; 2 School of Chemistry, Bharathidasan University, Tiruchirappalli, Tamil Nadu 620 024 India

**Keywords:** *D. gangeticum*, Soil chemistry, Pharmaco-chemical profiling, ADME-PK properties

## Abstract

**Background:**

*Desmodium gangeticum* (L.) DC. (Fabaceae) (DG) is a perennial non-climbing herb or shrub and folklore medicine, widely shows a large number of medicinal properties, as well as contains divergent bioactive compounds. Many of the herbal formulations contain this medicinal plant, which is considered as master of medicinal plant in Ayurveda. This study is an attempt to establish this plant material based on its pharmaco-chemical profiles with special reference to soil chemistry. The pharmaco-chemical features such as organoleptic, DNA sequence, physicochemical, proximate, phytochemical, UV, and FTIR profiling were carried out using standard techniques. Moreover, the ADME-PK properties of the selected molecules were established.

**Results:**

The pharmaco-chemical features like organoleptic, DNA sequence, physicochemical, proximate, phytochemical, UV, and FTIR profiling, ADME-PK properties, and soil chemistry of *D. gangeticum* revealed its unique and diagnostic peculiarities. DNA barcoding showed that the sequence was 99.77% similar to *D. gangeticum* (KP094638) having 100% query coverage. The soil analysis revealed the presence of moderately high content of NPK and sufficient amount of all essential macro- and micronutrients (S, Fe, Mn, Cu, Zn, and B). The phytochemical profiling showed that the ethanolic extract of the aerial part contained glycoside, amino acid, phenols, alkaloids, flavonoids, and coumarins, while the ethanolic root extract of the plant revealed the presence of glycoside, amino acid, phenols, alkaloids, flavonoids, coumarins, and triterpenoids. FTIR results indicated that the plant extracts are mainly rich in phenolic derivatives. ADME-PK properties of pterocarpan such as gangetin (**1a**), gangetinin (**1b**), desmocarpin (**1c**), and desmodin (**1d**) were found to pass the Lipinski, Ghose, Veber, and Egan rules, supporting the drug-likeliness.

**Conclusion:**

This is the first record of pharmaco-chemical profiling of *D. gangeticum* along with soil chemistry, and this information helps in the proper identification and future studies on this species.

**Graphic abstract:**

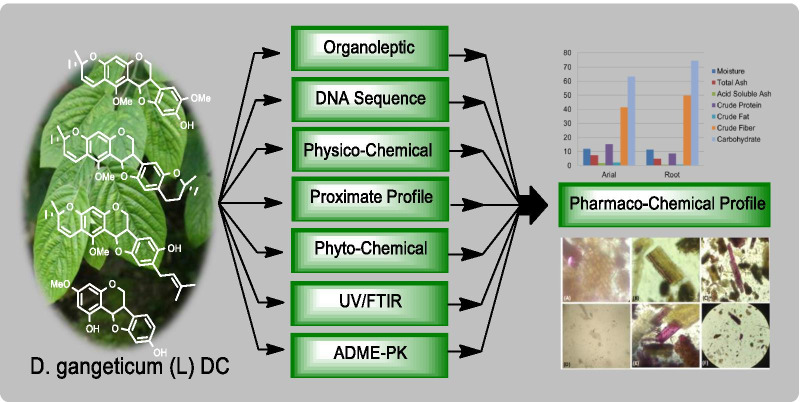

## Background

Recently, the importance of plant-based drugs and their therapeutic activities has high scope and has attracted great attention in the world [1]. The herbs contain various classes of complex molecules, which show good chemical biology [2]. A large population in many countries depends on herbal drugs for the treatment of several health problems [3]. In the present scenario, scientists have been focused to develop herbal formulations, especially to improve our immune system for preventing viral infections like the novel COVID-19 [4, 5]. The herbal formulation has various ingredients, which show great potential against various disorders [6–9].

Herbal drugs are a combination of complex or active mixtures of one or more plants. Lack of standardization and quality control of herbal drugs affects their actual potencies (efficiencies). Most of the drugs are in the form of crude extracts or powders which are a mixture of several components in the crude or processed state, and the active pharmacophore when isolated individually sometimes failed to give the desired action [10]. This implies that the pharmacological activity of the drugs is the synergistic effect of two or more components. The quality of herbal drugs depends on harvesting time and processing methods. There are few official guidelines for herbal formulation. Moreover, who are working on this field have their own parameters [11]. So, it is necessary to know the actual quality and purity of crude drugs before the formulation process. The standardization of herbal drug ingredients based on the pharmaco-chemical properties can lead to the dose determination, quality, and purity of drugs.

**Fig. 1:**
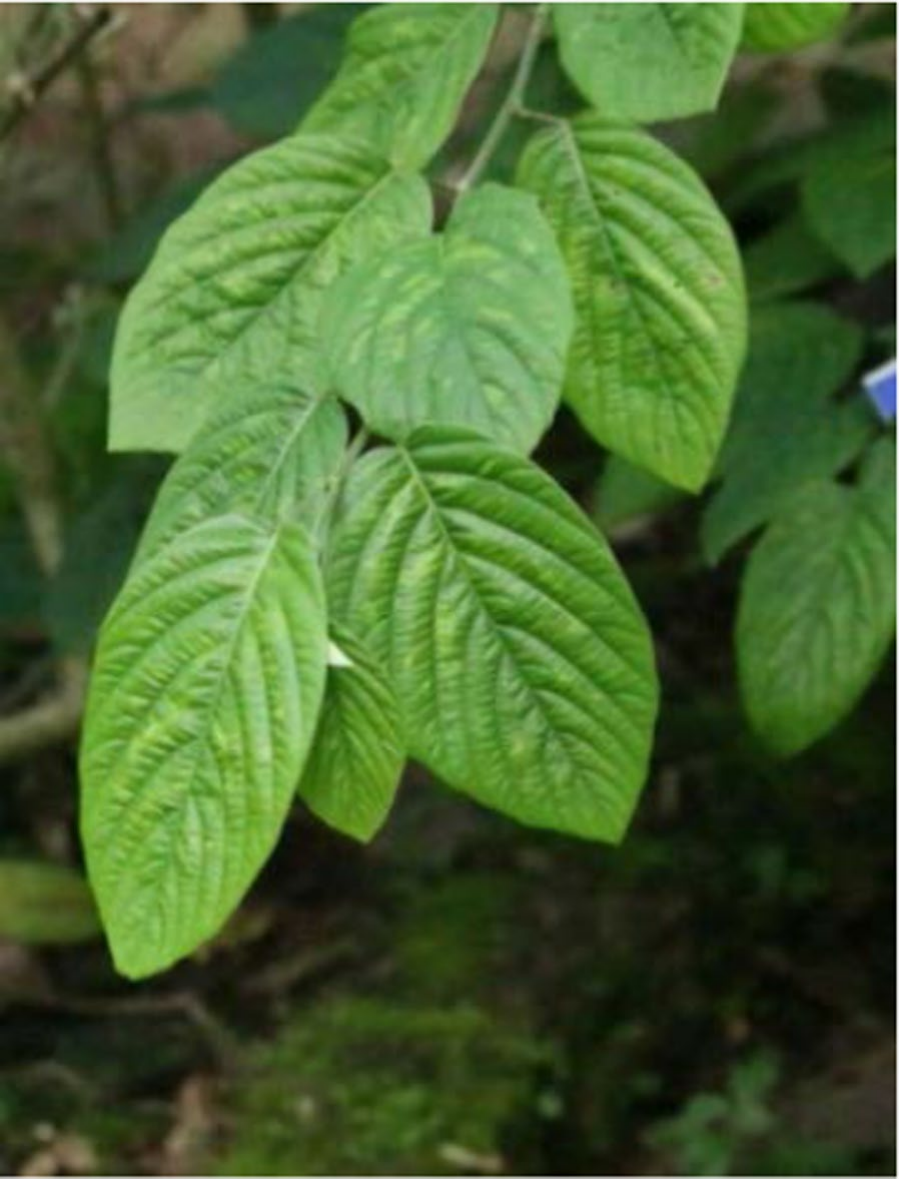
Wild photograph of *D. gangeticum* (L.) DC

*Desmodium gangeticum* (L.) DC. (Figure 1) belongs to the family Leguminosae (Fabaceae), subfamily Papillionaceae. The member of the genus *Desmodium* contains 170 tropical and subtropical species [12]. It is a perennial erect or ascending prostrate under shrub, distributed throughout the warmer parts of India. Matured plants are 60–130 cm high with angular branches, pubescent or glabrous. Leaves are simple, variable, ovate-oblong, or rounded in shape, 3–14 cm × 2–7 cm, acute to acuminate, glabrous above, pubescent beneath; petioles 1–2.5 cm long. Flowers are purple or white in color [13]. Many members of this genus have been used as traditional medicines [14], and chemical investigations have revealed the presence of isoflavones, isoflavanones, C-glycosyl flavonoids, pterocarpans, and coumaronochromones [13, 15, 16]. Looking into the species, *D. gangeticum* have exhibited many therapeutic properties.

In Ayurveda medicine, this plant species *D. gangeticum* is the chief of the ten ingredients in the “*Dasamula kwatha,”* which is used as a health tonic and pain reliever [17]. Moreover, in Unani medicine “*Arq dashmul*” contains this species roots extract and used against leucorrhoea and for pains due to cold [18]. From our preliminary field investigation, we found that many tribes from the Western Ghats region of Kerala used this plant for the treatment of various ailments such as fever, typhoid fever, cough, diuretic, inflammations, cough, asthma, and dysentery.

The high medicinal properties of this species have been attracting considerable interest in its industrial expansion. However, it made it difficult to meet the increasing market demand. Due to the increasing demand for herbal medicine, adulteration of genuine herbs with low-grade ones had increased significantly. [19]. It will affect the therapeutic efficiency of the drug. Therefore, it is a great interest to carry out a screening of *D. gangeticum* to validate their pharmaco-chemical profile. The standardization of herbal drugs is laid down for the pharmaco-chemical parameters. Depending on the geographic area and climate, the parameters may change [20]. In this aspect, here we disclosed the pharmaco-chemical analysis of *D. gangeticum* with special reference to soil chemistry.

## Methods

### Plant collection and processing

*Desmodium gangeticum* (L.) DC. was collected from the Idukki District of Kerala, India, during March–April 2019 (the atmospheric temperature found to be 25–33 °C with a humidity of 74%). The plant was identified by Dr. S. Soosairaj, Department of Botany, St. Joseph's College, Tiruchirappalli, Tamil Nadu, India (voucher number: 3006). Freshly collected plant material was cleaned to remove adhering dust, divided into different parts, and then dried under shade. The dried samples were powdered and stored at room temperature (25 °C) for further studies.

### Reagents and chemicals

All the reagents and chemicals used in this research work were procured from M/s Merck India, Ltd., Godrej One, 8th floor, Pirojsha Nagar, Eastern Express Highway, Vikroli East, Mumbai, Maharashtra 400079.

### DNA Barcoding

#### DNA extraction

Genomic DNA was extracted from young leaves of *D. gangeticum* using a modified cetyl trimethyl ammonium bromide (CTAB) method [21]. The quantity of DNA was measured using a NanoDrop Spectrophotometer (ND-2000, Thermo Scientific, Wilmington, USA), and the quality of DNA was checked via a 0.8% (w/v) agarose gel electrophoresis. The isolated DNA was stored at − 20 °C.

#### DNA amplification

The PCR was set with 50 μl reaction mixture containing 300 ng genomic DNA, 25 mM MgCl_2_, 1 mM dNTPs, 10 pmol rbcL primer (rbcL [R]: GTAAAATCAAGTCCACCRCG; rbcL [F]: ATGTCACCACAAACAGAAACTAAAGC), and 1 U Taq DNA polymerase (GeNet Bio, Daejeon, Korea). The PCR was performed using a master cycler (Eppendorf, Germany) with an initial denaturation at 94 °C for 5 min, followed by 35 cycles of 30 s denaturation at 94 °C, 45 s annealing at 54 °C, and 45 s extension at 72 °C, with a final extension at 72 °C for 5 min. The PCR products were resolved on a 1% (w/v) agarose gel at 70 V and stained with ethidium bromide. The agarose gel was photographed using the ChemiDoc XRS imaging system (Bio-Rad, Hercules, California, USA) to check the presence or absence of bands.

#### Sequencing and plant identification

The amplified products were used for DNA sequencing (sequencing PCR was performed in a final volume of 10 μl containing BigDye sequencing buffer (1X), template 40 ng (for 1000–2000 bp amplicon sequencing), primers (3.2 pM), and BigDye (0.5 μl)), and the sequences were processed with BioEdit software version 7.1.11 [22]. The chimeric artifacts were removed using the online tool Decipher [23]. The sequences were compared by using BLASTn (https://blast.ncbi.nlm.nih.gov/Blast.cgi) with the non-redundant database of sequences deposited at the NCBI GenBank database [24].

### Soil analysis

Soil samples were collected from the location of plant population grown (Idukki District of Kerala, India). The soil profile of different depths (0–10, 10–20, and 20–30 cm) was taken using the soil auger. Immediately after collection, soil samples were air-dried at room temperature (22 ± 1 °C), sieved (2 mm), and analyzed for different soil parameters. The dried sample (10 g) was used to evaluate the soil pH [25], electrical conductivity [26], total organic carbon (TOC) by titration method [27], and determination of total NPK and nitrogen [28]. Other elements (S, Fe, Mn, Cu, Zn, and B) are evaluated by spectrophotometric method [29] and flame photometer [30].

### Organoleptic and macroscopic evaluation

To carry out organoleptic evaluation, various sensory parameters of the plant material, such as color, odor, size, shape, and taste, were studied [31].

### Powder microscopy

The dried powdered aerial parts of *D. gangeticum* were studied by placing small amounts of powder on a slide and observing under the microscope. Samples were mounted on 5% glycerin solution or stained with reagents such as N/50 iodine for the observation of starches or 0.1% w/v phloroglucinol plus a drop of concentrated hydrochloric for the observation of lignified cells [32]. Characteristic structures and cell contents such as fibers, vessels, cork cells, calcium oxalate crystals, and plant cells were observed at various magnifications, and photomicrographs were taken.

### Physicochemical analysis

The dried powdered aerial parts and root were used for determination of physicochemical contents, viz. total ash, acid-soluble ash, water-soluble ash, and alcohol-soluble as hand extractive values using different solvents, viz. ethanol, chloroform, ethyl acetate, hexane, toluene, petroleum ether, and water [19, 33].

### Proximate analysis

The dried powdered aerial parts and root were used for evaluation of proximate contents through total ash, crude fiber, crude protein, carbohydrate, crude fat, dry matter, and moisture content [34].

### Preparation of extracts

The dried aerial/root (100 g) was powdered in a plant sample grinder at a controlled temperature and used for extraction using suitable solvents in a Soxhlet extraction apparatus attached with a rotary vacuum evaporator (Buchi, Switzerland). Solvents were removed using a rotary vacuum evaporator at 175 mbar at a controlled temperature.

### Phytochemical analysis

Preliminary phytochemical screening was done as per the standard procedure [35] for various phyto-constituents such as steroids (Liebermann–Burchard test), terpenoids (Salkowaski test), alkaloids (Mayer’s test), tannins (ferric chloride test), flavonoids (alkaline reagent test), carbohydrates (Benedict’s test), and amino acids (Biuret test).

### UV and FTIR profiling

The UV spectra of the ethanol extracts of the aerial and root were recorded on a Jasco UV spectrophotometer at a wavelength range of 200–600 nm with a scan speed of 400 nm/min. The spectra used in obtaining the structural properties of the selected plant extract were obtained from the Fourier-transform infrared spectrometer equipped with an attenuated total reflectance (ATR-FTIR), model PerkinElmer Spectrum 400. In the ATR-FTIR method, the sample to be analyzed is placed directly into the sample cell, where a good and reproducible contact between the sample and the crystal of reflection is obtained nondestructively, producing good-quality infrared spectra. The FTIR spectra were recorded in the range of 4000–700 cm^−1^.

### ADME properties

Pharmacokinetics and drug-likeness prediction for the compounds 1a-d were performed by online tool SwissADME [36] of the Swiss Institute of Bioinformatics (http://www.sib.swiss) [37]. 2D structural models were drawn in ChemBioDraw Ultra version 15.0 (Cambridge Software), and SMILES of 1a-d was translated into molfile by online SMILES translator and structure file generator found in online tool SwissADME. The analysis task was done to check whether the compound was an inhibitor of isoforms of the Cytochrome P450 (CYP) family, such as CYP1A2, CYP2C19, CYP2C9, CYP2D6, and CYP3A4. Also, pharmacokinetics (such as gastrointestinal absorption, P-glycoprotein, and blood–brain barrier) and drug-likeness prediction was done, such as Lipinski, Ghose, and Veber rules and bioavailability score [38–40]. The Lipinski, Ghose, Egan, Mugges, and Veber rules were applied to assess drug-likeness to predict whether a compound is likely to be bioactive according to some important parameters such as molecular weight, Log P, number of HPA, and HBD. The SwissADME tool used a vector machine algorithm (SVM) [41] with fastidiously cleaned large datasets of known inhibitors/non-inhibitors as well as substrates/non-substrates.

## Results

The chemical profiling of soil is recorded in Table 1. It is found that pH of the soil is strongly acidic (pH = 5.21) in nature and electrical conductivity was 0.12 dSm^−1^ and a very high content of organic carbon (1.52%) was detected. The NPK analysis revealed moderately high content of nitrogen (N = 446.7 kg/ha) and very high content of phosphorus (P = 444.7 kg/ha) and potassium (K = 313.4 kg/ha) in the soil. Furthermore, sulfur (S) analysis showed a sufficient amount of 6.5 ppm. Moreover, micronutrient analysis revealed sufficient contents of iron (Fe, 18.3 ppm), manganese (Mn, 18.7 ppm), copper (Cu, 6.17 ppm), zinc (Zn, 9.95 ppm), and boron (B, 2.10 ppm).

**Table 1:** Soil analysis

Parameters	Range
pH	5.21
Electrical conductivity (EC)	0.12 dSm^−1^
Organic carbon (C)	1.52%
Nitrogen (N)	446.7 kg/ha
Phosphorus (P)	105.4 kg/ha
Potassium (K)	313.4 kg/ha
Sulfur (S)	6.5 ppm
Iron (Fe)	18.3 ppm
Manganese (Mn)	18.7 ppm
Copper (Cu)	6.17 ppm
Zinc (Zn)	9.95 ppm
Boron (B)	2.10 ppm

The macroscopic and organoleptic evaluation is an easy method and is carried out at the time of plant collection. The various observed features of the plant parts were recorded and are depicted in Table 2.

**Table 2:** Organoleptic studies of *D. gangeticum*

Parameter	Organoleptic evaluation		
	Leaf	Stem	Root
Taste	Sweet	Characteristic	Sweet
Odor	Characteristic	Characteristic	Characteristic
Color—upper/outer	Dark green	Light brown	Reddish brown
Color—lower/inner	Light green	Yellowish green	Light brown
Texture of powder	Coarse	Sandy	Coarse
Shape	Ovate-oblong	Angular	*-*
Surface	Hairy	Glabrous	Smooth

**Fig. 2:**
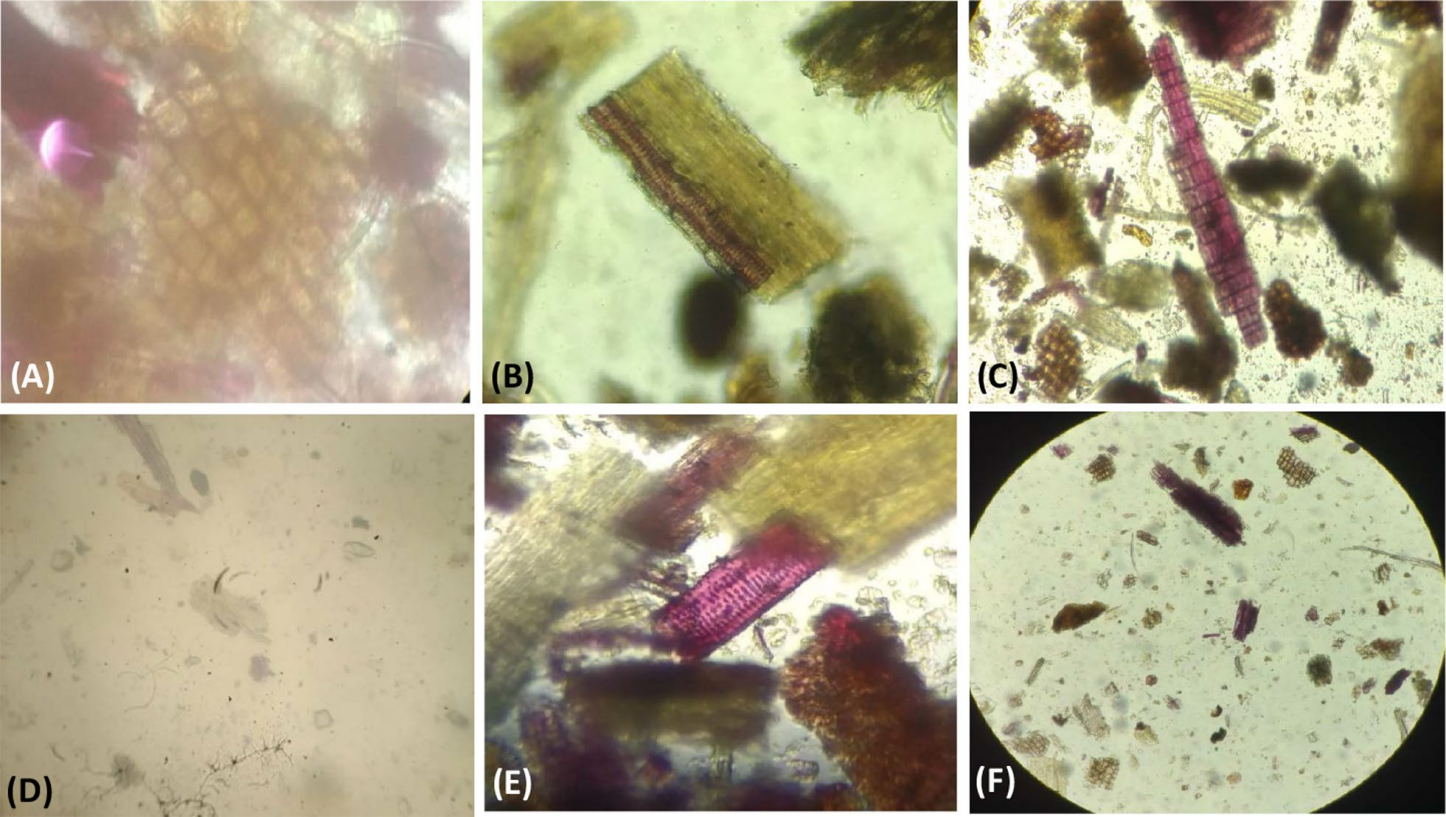
Powder microscopy of *D. Gangeticum.* **A** Epidermis (× 40), **B** tangentially elongated cork cells (× 40), **C** separated fiber (× 40), **D** prismatic crystals of calcium oxalate (× 40), **E** pitted vessel (× 40),** F** simple fibers (× 40)

The result of powder microscopy (Fig. 2) reveals the presence of various parts like epidermis (A), tangentially elongated cork cells (B), separated fiber (C), prismatic crystals of calcium oxalate (D), pitted vessel (E), and simple fibers (F). Moreover, we did not find any symptoms of nutrient deficiency, like stunted growth, death of plant tissue, or yellowing of the leaves caused by a reduced production of chlorophyll, a pigment needed for photosynthesis. Additionally, the root structure was found to be normal.

Furthermore, we have used the ribulose-1,5-bisphosphate carboxylase/oxygenase large subunit (*rbcL*) molecular marker for plant species identification. The NCBI BLAST results showed that the sequence was 99.77% similar to *D. gangeticum* (KP094638), and also it showed 100% query coverage.

**Fig. 3:**
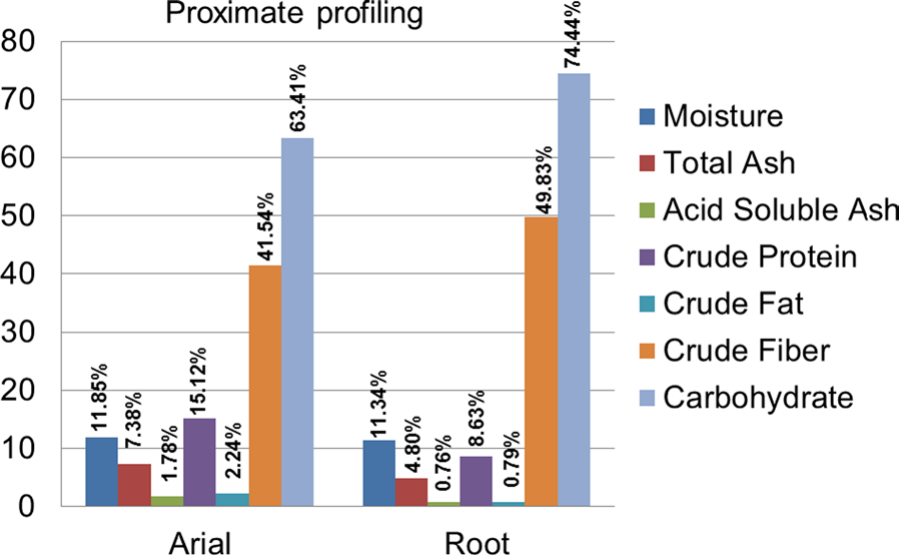
Proximate profiling of crude powder of *D. gangeticum* (L.) DC.

The physicochemical parameters such as percentage of moisture content, total ash, and acid insoluble ash were found to be 11.85%, 7.38%, and 1.78% in areal and 11.34%, 4.80%, and 0.76% for root, respectively (Fig. 3). The successive Soxhlet extractive yields obtained from water, followed by ethanol, ethyl acetate, and hexane, are calculated (Table 3).

**Table 3:** Extractive yield analysis

Extract	Yield (%)^a^	
	Aerial	Root
Ethanol	5.8 ± 0.01	4.80 ± 0.02
Water	8.8 ± 0.12	7.20 ± 0.10
Hexane	2.9 ± 0.01	2.50 ± 0.02
Ethyl acetate	3.2 ± 0.03	2.60 ± 0.01

^a^Mean of 3 readings ± SEM

The proximate parameters such as percentage of crude fiber, crude protein, crude fat, and carbohydrate contents of the aerial and root are also evaluated and are shown in Figure 3. Aerial part has a high content of crude fiber of 11.85%, crude protein of 15.12%, crude fat of 2.24%, and carbohydrate of 63.41%. Root powder has protein of 8.63% and crude fat of 0.73%. Moreover, the crude fiber (49.83%) and carbohydrate (74.44%) were found to have the highest percent.

The results of the phytochemical profiling (Table 4) showed that the ethanolic extract of the aerial part contained glycoside, amino acid, phenols, alkaloids, flavonoids, and coumarins, while the ethanolic root extract of the plant revealed the presence of glycoside, amino acid, phenols, alkaloids, flavonoids, coumarins, and triterpenoids.

**Table 4:** Phytochemical screening of ethanolic extracts of aerial and root

Phytochemical compounds	Aerial	Root
Tannins	−	−
Glycoside	+	+
Amino acid	+	+
Phenols	+	+
Volatile oils	−	−
Alkaloids	+	+
Saponins	−	−
Flavonoids	+	+
Coumarins	+	+
Phytosterols	−	−
Triterpenoids	−	+

(+), present; (−), absent

**Fig. 4:**
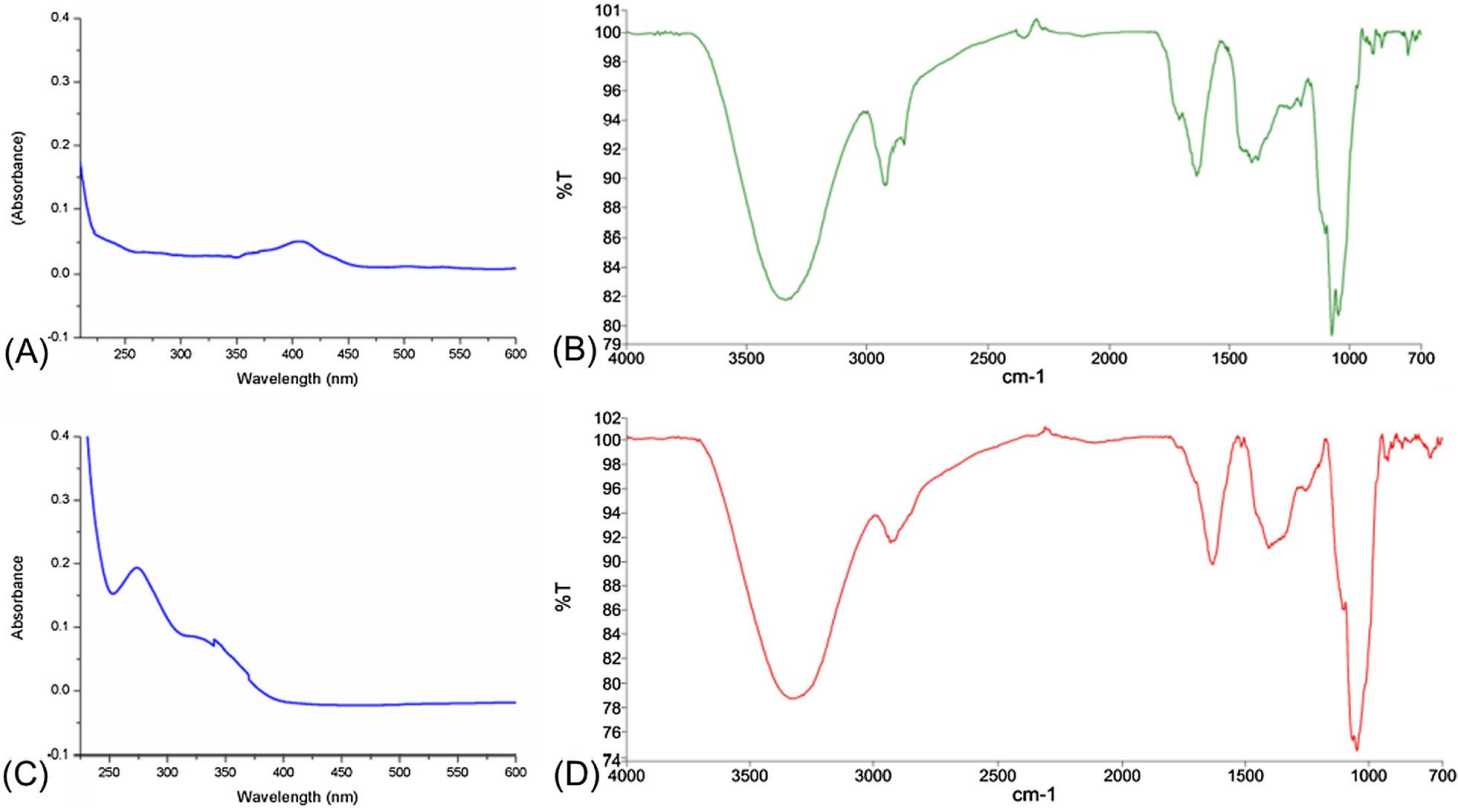
UV and FTIR profiling of EtOH extracts; aerial (**A**, **C**); root (**B**, **D**)

The UV spectra of the ethanolic extract of aerial (A) and root (C) are shown in Fig. 4. The FTIR analysis (B/D) shows a broad band at around 3311 and 2918 cm^−1^ which can be attributed to O–H group present in phenols. These results indicated that the plant extracts are mainly rich in phenolic class of derivatives.

**Fig. 5:**
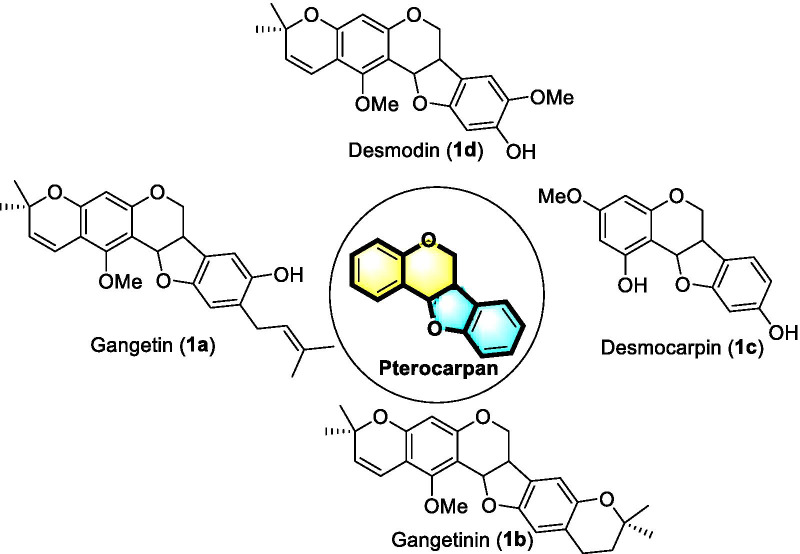
Selected pterocarpan derivatives from *D. gangeticum* (L.) DC.

The ADME-PK properties of selected pterocarpans (**1a**–**1d**, Fig. 5) are recorded in Tables 5 and 6. It was shown that all the compounds have high gastrointestinal (GI) absorption, good blood–brain barrier (BBB) permeability, and also P-glycoprotein (P-gp) permeability. The lipophilicity (log P_o/w_) and skin permeation (log Kp) of compounds were observed in the range of 1.64–4.81 and 4.99–6.87 cm/s, respectively.

**Table 5:** Pharmacokinetic studies of pterocarpans

Pharmacokinetics properties										
GI absorption		BBB permeation	Log *p*_o/w_	P-*gp*	Inhibition of cytochrome P450					Log *Kp*
					CYP1A2	CYP2C19	CYP2C9	CYP3A4	CYP2D6	
**1a**	High	Yes	4.81	Yes	×	√	√	×	√	− 4.99 cm/s
**1b**	High	Yes	4.71	Yes	×	√	√	√	√	− 5.41 cm/s
**1c**	High	Yes	1.64	Yes	×	×	×	√	×	− 6.87 cm/s
**1d**	High	Yes	3.36	Yes	√	√	√	√	√	− 6.15 cm/s

**1a**, gangetin; **1b**, gangetinin;, **1c**, desmocarpin; **1d**, desmodin; GI, gastrointestinal absorption; BBB, blood–brain barrier permeability; Log *P*_o/w_, lipophilicity; P-*gp*, P-glycoprotein substrate; Log *Kp*, skin permeation

**Table 6:** Drug-likeness and medicinal chemistry of pterocapans

Drug-likeness and medicinal chemistry											
Log *s*		Drug-likeness					Bioavailability	Medicinal properties			Synthetic accessibility
		Lipinski	Ghose	Veber	Egan	Mugge		TPSA (*A*^2^)	PAINS (alert)	Lead likeness	
**1a**	− 5.98	√	√	√	√	×	0.55	57.15	0	No^a^	4.94
**1b**	− 5.74	√	√	√	√	√	0.55	46.15	0	No^a^	4.90
**1c**	− 2.82	√	√	√	√	√	0.55	68.15	0	Yes	4.38
**1d**	− 4.60	√	√	√	√	√	0.55	66.38	0	No^a^	4.57

**1a, **gangetin; **1b,** gangetinin;**, 1c,** desmocarpin; **1d,** desmodin; Log *s*, solubility class; TPSA, topological polar surface area; PAINS, pan-assay interference structure

^a^Molecular weight < 350

Furthermore, compounds **1a**–**d** showed inhibition of cytochrome P450 isomers. Also, topological polar surface area (TPSA) of **1a**–**c** was found to have 46.15–68.15 Å (≤ 140 Å), indicating that compounds have appropriate oral bioavailability (0.55). Moreover, all compounds **1a**–**d** meet the criteria of drug-likeness assessment based on Lipinski, Ghose, Veber, and Egan rules. The drug lead-likeness shows that compound desmocarpin (**1c**) is the most druggable substance without any violation. A combination of fragment contributions and a complexity penalty of **1a**–**1d** indicated good synthetic accessibility.

## Discussion

The pharmaco-chemical constituent in the crude drug is greatly influenced by geographic–climatic conditions, and external conditions such as light intensity, water availability, and soil composition affect both the quantitative and qualitative composition of metabolites [42]. Moreover, this might influence the chemical biology of drugs. The plant growth and development largely depend on the combination and concentration of mineral nutrients available in the soil [43]. A deficiency of any one of them may affect plant physiological functions, and it leads to changes in the pharmaco-chemical properties [44, 45]. Based on this aspect, we began pharmaco-chemical characterization of *D. gangeticum* with special reference to soil chemistry and geography.

Nutrients are considered essential for plants [46]; macronutrients are the building blocks of crucial cellular components like proteins and nucleic acids; as the name suggests, they are required in large quantities. Nitrogen (N), phosphorus (P), magnesium (Mg), and potassium (K) are some of the most important macronutrients [47]. Carbon (C), hydrogen (H), and oxygen (O) are also considered macronutrients as they are required in large quantities to build the larger organic molecules of the cell [48].

The nutrients are usually obtained from the soil through plant roots. The soil nature and lack of nutrient availability in soil can make harder for plants to absorb required nutrients. In general, the soil pH is close to neutral and many plants grow successfully in a soil pH range of 5.5 to 7.5. The availability of plant nutrients is significantly affected by soil pH [49]. The soil of this region generally shows acidic in nature, high in nitrogen, and poor in bases; the texture is dark reddish-brown to black with loamy to silty loam [50]. In our present study, it was found that the soil pH was 5.21 and the electrical conductivity was 0.12 dSm^−1^. The NPK analysis revealed moderately high content of nitrogen and very high content of phosphorus and potassium in the soil. Furthermore, sulfur content was found to be sufficient. Moreover, micronutrient analysis revealed sufficient contents of iron (18.3 ppm), manganese (18.7 ppm), copper (6.17 ppm), zinc (9.95 ppm), and boron (2.10 ppm). These micronutrients are required in very small amounts and often required as cofactors for enzyme activity.

We have taken a closer look at the above values and concluded that a sufficient amount of all essential macro- and micronutrients is present in the soil. It helps with normal plant growth and development. These studies did not find any symptoms of nutrient deficiency, like stunted growth, death of plant tissue, or yellowing of the leaves caused by a reduced production of chlorophyll, a pigment needed for photosynthesis. Additionally, the root structure was found to be normal. In general, nutrient-limited soils are a change in root structure that may increase the overall surface area of the root to increase nutrient acquisition or may increase elongation of the root system to access new nutrient sources [51].

In herbal formulation, the proper identity of many drug species is important [52]. A single plant species has several different commercial or medicinal names in different regions. However, authentication of the botanical identity and ascertaining the genuineness of drug is a great concern in practical situation. To some extent, it can be overcome by drug characterization. This is done by estimating their active principles [53]. There are many advanced approaches available to authenticate botanical drugs, ranging from simple morphological examination to physical and biochemical analysis, chromatographic, and molecular techniques [54]. Among these, powder microscopy is the most practical method for primary authentication [55], and it is a unique, valuable, rapid, and cost-effective assessment tool and plays an important role in the authentication and assessment of herbal plants [56]. The powder microscopy reveals the presence of various parts such as epidermis, tangentially elongated cork cells, separated fiber, prismatic crystals of calcium oxalate, pitted vessel, and simple fibers. These features are useful in identifying species with similar morphological characters.

DNA barcoding has been a powerful tool for plant species identification in recent years due to its simplicity and high accuracy as compared to the complexity and subjective biases associated with morphology-based identification of taxa. This technique is reliable and is not affected by external factors such as climates, age, or plant part [57]. We have used the ribulose-1,5-bisphosphate carboxylase/oxygenase large subunit (*rbcL*) molecular marker for plant species identification. The results showed that the sequence was 99.77% similar to *D. gangeticum* with 100% query coverage. Previously, the *rbcL* markers were successfully used to identify many medicinal plants [58, 59]. Therefore, results also provide the *rbcL* as an important universal molecular marker for medicinal plant identification.

In our preliminary field investigation, it is revealed that this plant is used to cure various ailments in the tribal people communities of the Western Ghats region of Kerala; therefore, interest was developed to know other possible health benefits of this plant. Besides using such plants as a crude drug, proximate profiling can be essential. The proteins, fats, and carbohydrates are important nutrients to be assessed in the medicinal plant. These proteins enclose essential amino acids and have nutritional values for human health [60].

The physicochemical parameters such as percentage of moisture content, total ash, and acid insoluble ash were established. The successive Soxhlet extractive yields obtained from water, followed by ethanol, ethyl acetate, and hexane, are calculated. In the future, the optimal ash and extractive values help to identify the inorganic substance or impurities present along with the crude drug. Overall, this leads to quality improvement in the drug.

The proximate parameters such as percentage of crude fiber, crude protein, crude fat, and carbohydrate contents of the areal and root are also evaluated, and the areal part has a high content of crude fiber, crude protein, crude fat, and carbohydrate. The root part was found to have only less content of protein (8.63%) and crude fat (0.73%). The crude fiber (49.83%) and carbohydrate (74.44%) were found to have the highest percent. These findings help to set up certain standards for crude drugs and the determination of nutritive values. Reports suggest that medicinal plant species have their nutrient/proximate composition besides having bioactive constituents. These nutrients are essential for the physiological functions of the human body [61].

The results of the phytochemical profiling showed that the ethanolic extract of the aerial part contained glycoside, amino acid, phenols, alkaloids, flavonoids, and coumarins, while the ethanolic root extract of the plant revealed the presence of glycoside, amino acid, phenols, alkaloids, flavonoids, coumarins, and triterpenoids. Moreover, our FTIR analysis gave an idea about the plant extracts mainly rich in phenolic derivatives.

The compounds such as gangetin (**1a**), gangetinin (**1b**), desmocarpin (**1c**), and desmodin (**1d**) are belonging to pterocarpan class of phenolic derivatives which was reported from the species *D. gangeticum* [13]. The pterocarpans constitute the second largest group of natural isoflavonoids and show many biological properties [62]. The main structural feature of pterocarpans consists in the presence of a tetracyclic system of benzofuran–benzopyran rings [63].

In this context, we tried to understand the absorption, distribution, metabolism, and excretion (ADME) properties of pterocarpans (**1a**–**1d**), through a silicon study. The computer modeling of ADME properties of compounds provides an idea about structure–property relationships and drug metabolism and pharmacokinetics properties based on the compound structure [64]. Results revealed that all pterocarpans (**1a**–**1d**) showed high gastrointestinal (GI) absorption and good blood–brain barrier (BBB) permeability, and also compounds have P-glycoprotein (P-gp) permeability. The lipophilicity (log P_o/w_) and skin permeation (log Kp) of compounds were observed in the range of 1.64–4.81 and 4.99–6.87 cm/s, respectively. Furthermore, compounds **1a**–**d** showed inhibition of cytochrome P450 isomers. Topological polar surface area (TPSA) is the sum of polar atoms present in the molecule (**1a**–**c**) surface, and it helps to predict drug transport qualities such as intestinal absorption and BBB penetration. The TPSA of **1a**–**c** was found to be 46.15–68.15 Å (≤ 140 Å). This value indicates good blood–brain barrier penetration. Moreover, all compounds **1a**–**d** meet the criteria of drug-likeness assessment based on Lipinski, Ghose, Veber, and Egan rules. The drug lead-likeness shows that compound desmocarpin (**1c**) is the most druggable substance without any violation. A combination of fragment contributions and a complexity penalty of **1a**–**1d** indicated good synthetic accessibility.

## Conclusion

The pharmaco-chemical features like organoleptic, DNA sequence, physicochemical, proximate, phytochemical, UV, and FTIR profiling, ADME-PK properties, and soil chemistry of *D. gangeticum* revealed its unique and diagnostic peculiarities. DNA barcoding showed that the sequence was 99.77% similar to *D. gangeticum* (KP094638) having 100% query coverage. The soil analysis revealed the presence of moderately high content of NPK and sufficient amount of all essential macro- and micronutrients. The phytochemical profiling showed important class of phytoconstituents in the aerial and root parts of the plant. FTIR profiling indicates that the plant extracts are mainly rich in phenolic derivatives. Further, ADME-PK properties of selected pterocarpan such as gangetin (**1a**), gangetinin (**1b**), desmocarpin (**1c**), and desmodin (**1d**) were found to pass the Lipinski, Ghose, Veber, and Egan rules, supporting the drug-likeliness. This useful information helps future works on this species.

## Data Availability

Data and material are available upon request.

## Abbreviations

ADME-PK: Absorption, distribution, metabolism, excretion—pharmacokinetics
CTAB: Cetyltrimethylammonium bromide
PCR: Polymerase chain reaction
ATR-FTIR: Attenuated total reflectance Fourier-transform infrared spectrometer
SMILES: Simplified molecular-input line-entry system
CYP: Cytochrome P450
GI: Gastrointestinal absorption
BBB: Blood–brain barrier
TPSA: Topological polar surface area
PAINS: Pan-assay interference structure

## Declarations

### Plant authentication

Healthy and disease-free plants of *D. gangeticum* were collected from the Idukki District of Kerala, India, during March–April 2019. The collected specimens were identified by using DNA barcoding, and the sequences were compared by using BLAST results. The confirmed sequences were submitted to the NCBI GenBank, and the sequence was given an accession number MW021162.
